# Cerebellar ganglioglioma

**Published:** 2012-05-22

**Authors:** Badr Fedoul, Zouhayr Souirti

**Affiliations:** 1 Hassan II University hospital, Fez, Morocco

**Keywords:** Ganglioglioma, cerebellar vermis, histology, tumour, Morocco

## Abstract

The cerebellar location of ganglioglioma (GG) is exceptional. We report one case of a 27-year-old man who underwent an intracranial hypertension syndrome and a static cerebellar syndrome. Brain magnetic resonance images revealed a cyst image in the vermis. Histological study after surgical removal, revealed a ganglioglioma tumor. Through this case and literature review, the authors discuss some epidemiological, histological, clinical, radiological and management features of this very rare tumor.

## Introduction

Gangliogliomas occur frequently in temporal lobe, and induce incontrollable epilepsy. GG is very rare in cerebellum with 31 cases reported previously [1]. The main elements found in gangliogliomas are atypical neurons, astrocytes, and a fibrovascular stroma. In this communication we report a rare case of GG in the cerebellar hemisphere which was radiologically diagnosed as a pilocystic astrocytoma.

## Patient and case report

**Figure 1:**
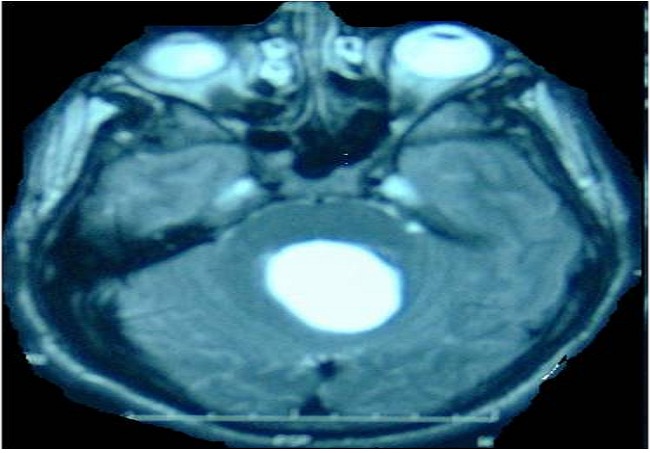
Brain MRI T2, axial sequence, showing a hyperintense cyst image in the vermis. The cyst produces a mass effect on brainstem and 4th ventricle

**Figure 2:**
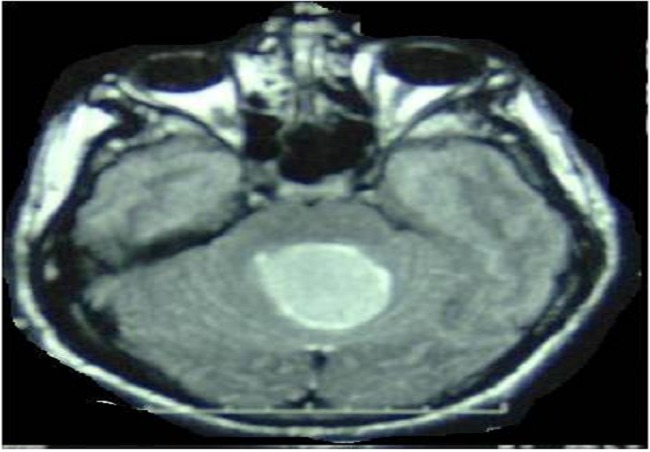
Brain MRI FLAIR, axial sequence, showing a hyperintense signal in the cyst different to CSF signal

**Figure 3:**
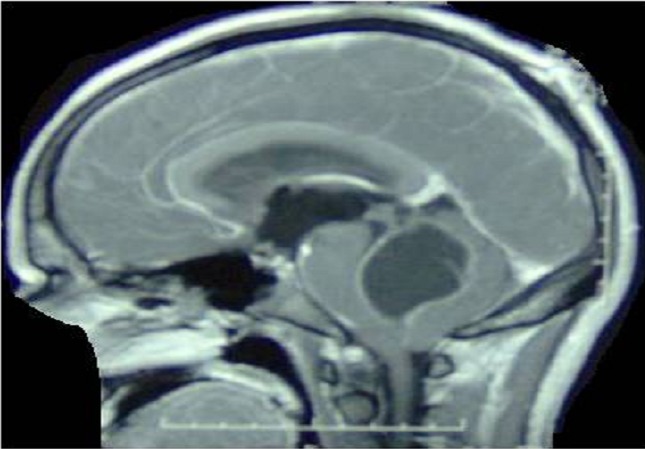
Brain MRI T1 sagittal sequence after Gadolinium injection showing homogeneous wall enhancement

A 27-year-old man complained of diffused headache for 5 months with some episodes of vomiting. There was no significant family history. On neurological examination, there was a static cerebellar syndrome associated with a papilloedema. Brain magnetic resonance (MR) images revealed a cyst lesion in the vermis with ring enhancement after gadolinium administration (Figure 1, Figure 2, Figure 3). The tumor was associated with obstructive hydrocephalus. The cystic portion of tumor showed the cerebrospinal fluid-like signal intensity on T1, T2 MR images but not in FLAIR images (Figure 2). There was no calcification within the tumor on the computed tomography (CT) scan. We performed the operation via a right suboccipital craniectomy. After opening of tensed dura, a cyst was noted and 20 ml of xanthochromic fluid was then aspirated. The wall was totally removed macroscopically. In histological exam, the wall contained atypical neurons and astrocytes and a fibrovascular stroma. Postoperatively, there was a complete recovery. No further treatment was given. At last follow-up, 24 months after surgery; this man had no problems in daily life.

## Discussion

Ganglioglioma is the most commonly encountered glial-neuronal neoplasm of the central nervous system, with its incidence of 0.4% to 4.3% [2]. This tumor is usually seen in children and young adults and there is no gender preponderance [3]. In the series by Chang and associates, of 133 posterior cranial fossa tumors, only 1 case of GG was described. Courville (1930) correctly acknowledged the mixed histological composition of mature ganglion cells and glial cells of varying proportions and degrees of differentiation.

Both components usually exhibit low grades of malignancy, but it is the grade of the glial element that predicts the biological behavior. GG is part of the family of mixed glial ganglion cell tumors that are included within grade I or II of the WHO classification; however, occasional cases of anaplastic GG (WHO grade III) have been reported [4]. Malignant degeneration is also rare, with an estimated incidence of 6%. Although anaplastic transformation of both the glial and neuronal components has been reported, malignant changes appear to be confined mostly to the glial element of the tumor [5] Leptomeningeal and subarachnoid spread is uncommon but may occur [6].

There are no specific clinical findings to indicate cerebellar GG and to discriminate that from other cerebellar lesions. Cerebellar GGs have a generally short mean history (1.6 years) [7] GG may be revealed by static or kinetic cerebellar syndrome and intracranial hypertension syndrome due to obstructive hydrocephalus.

Clinical presentation of infratentorial GG varies depending on the structures involved. Associated symptoms include cranial nerve deficits (hearing loss, intractable facial pain, hemifacial seizures), hemiparesis, gait disturbance, and headache [8]. There are some reports describing epilepsy of cerebellar origin in patients with cerebellar GG. Whether the cerebellar tumor either initiates the seizure or lowers their threshold is still being debated.

Although the exact mechanism for the epilepsy arising in the cerebellum is not known, invasive electrophysiological monitoring with depth electrodes has confirmed the cerebellum as the site of seizure origin. It is thought that the seizure arises in the cerebellum and may generalize secondarily, spreading throughout the cortical surface. Surgical resection of GG has resulted in remission of the seizures in all reviewed cases [9]. Radiologically the cyst with an enhancing mural nodule is classic, but not specific for GGs. The tumor is often isodense or hypodense in the precontrast CT, with calcification present in 6% to 30%. On MR imaging studies, GGs are usually hyperintense on T2-weighted images and isointense to slightly hypointense on T1-weighted images. The variable signal in cystic portion depends on whether the contents are proteinaceous, hemorrhagic, or contain cerebrospinal fluid [2].

Gadolinium enhancement of the solid component of the GGs is observed in about half of the cases, although the pattern varies from intensely homogenous to heterogeneous. Although there is considerable variability in the radiographic appearances of the cerebellar GGs, almost all tumors appear as a discrete, solid/cystic lesion with mild mass effect and little or no surrounding edema [10].

The main differential diagnosis of cerebellar GG in children or young adults is typically the pilocytic astrocytoma [1]. Surgery is the treatment of choice for cerebellar ganglioglioma. Complete resection leads to a good long-term prognosis which results in greater than 90% of 5-year survival. However, because of the location and vicinity to vital and sensitive brain areas and their adherence to the tumor, complete resection of infratentorial GG is often not possible without producing severe deficits or even death, thus leaving only space for subtotal resection [11].

Nevertheless, the diagnosis of GG is of great importance because of its comparatively better prognosis than other tumors in this location. Radiotherapy is not recommended in management of GG because of the entirely radio-resistant nature. The radiotherapy doesn't affect the poetentiel growth of GG [2]. Some reports suggest that postoperative radiation may predispose a GG to malignant degeneration [12].

## Conclusion

The cerebella location of ganglioglioma is exceptional and a diagnostic surprise for the neurosurgeon. It is usually seen in children and young adults. Complete resection is the treatment of choice. Even with partial resection, prognosis remains favorable. Radiotherapy is not required.
